# Downlook Technique for Superior (Head-End) Phacoemulsification in Deep-Set Eyes

**DOI:** 10.7759/cureus.115218

**Published:** 2026-08-26

**Authors:** R Balamurugan, Kiratmeet Singh, Shubhangi SN Prasad, Shagila R, Mishika Jain

**Affiliations:** 1 Ophthalmology, All India Institute of Medical Sciences, Madurai, Madurai, IND; 2 Ophthalmology, All India Institute of Medical Sciences, Mangalagiri, Mangalagiri, IND

**Keywords:** cataract surgery, deep-set eyes, downlook approach, phacoemulsification, prominent brow, prominent supraorbital rim, superior approach, superior clear corneal incision

## Abstract

Deep-set eyes present a technical challenge during phacoemulsification cataract surgery because restricted access to the superior globe limits instrument maneuverability, particularly when surgery is performed through a superior (head-end) approach. Although the temporal approach is commonly preferred in such cases, a superior approach may be indicated in selected situations based on corneal pathology, astigmatism, or surgeon preference. We describe a simple "downlook technique" to facilitate superior (head-end) phacoemulsification in a 67-year-old man with bilateral Grade III-IV nuclear cataracts and deep-set eyes who underwent left-eye phacoemulsification with posterior chamber intraocular lens implantation under topical anesthesia. During surgery, the patient was instructed to maintain a downward gaze whenever superior access was required, thereby improving exposure of the superior corneal region and facilitating the creation of the main corneal incision, capsulorhexis, phacoemulsification, intraocular lens implantation, and main wound hydration. The remaining surgical steps were performed in the primary gaze when adequate maneuverability was available. The surgery was uneventful, and the patient achieved a best-corrected visual acuity of 6/6 on the first postoperative day with a clear cornea and a well-centered intraocular lens. The downlook technique provides a simple, practical method to improve superior globe exposure without altering the patient's body position, making it a useful adjunct during superior (head-end) phacoemulsification in patients with deep-set eyes, provided adequate patient cooperation is achieved.

## Introduction

Deep-set eyes have not been clearly defined in the literature but are generally described as deeply positioned globes within the orbits, often associated with a deep superior sulcus, a prominent brow ridge, and a prominent nasal bridge [1,2]. Phacoemulsification in patients with deep-set eyes is technically challenging because the deeply positioned globe provides limited space for surgical instrument movement. In the left eye, the nasal bridge further restricts instrument maneuverability, particularly in the superior and superonasal regions. As a result, introducing surgical instruments through the corneal incision in a horizontal plane becomes difficult, often requiring a steeper angle of entry that is less ergonomic and more technically demanding. To overcome this difficulty, many surgeons prefer a temporal approach [3], as it provides easier access. Some surgeons may avoid the temporal approach because of increased side-to-side head movement during surgery. Furthermore, the lack of eyelid support may predispose to wound leakage and increase the risk of postoperative infection [3]. Some surgeons are more comfortable operating from the superior (head-end) position because of familiarity and surgical preference.

In the superior approach, another option to achieve better exposure of the superior globe is the Trendelenburg position [2,4-6], in which the patient lies supine with the feet elevated. This position may facilitate inferior rotation of the globe while maintaining alignment with the operating microscope. However, the Trendelenburg position has been associated with increased intraocular pressure and other ocular and systemic complications, making it less desirable for intraocular surgery, except in some spine-related procedures [4-6]. A reverse Trendelenburg position with the neck extended [2] favors optimal exposure of the superior globe and may help avoid complications associated with the Trendelenburg position. Nevertheless, achieving adequate globe orientation in this position often requires extension of the patient's neck, which can be uncomfortable, particularly in elderly patients or those with cervical spine disorders.

To achieve a similar orientation of the globe without altering the patient's body position, we adopted a new approach called the downlook technique. In this technique, the patient is instructed to maintain a downward gaze during the intraocular procedure, thereby improving exposure of the superior limbus and conjunctiva for superior or superonasal main-port incision creation and facilitating surgical instrument maneuverability. The degree of downward gaze can be adjusted intraoperatively to optimize the surgeon's visualization and comfort. If required, slight chin elevation can further improve exposure. Both the degree of downward gaze and chin elevation should be adjusted to provide optimal visualization and retroillumination. In this case report, we describe the downlook technique as a simple method to facilitate superior (head-end) phacoemulsification under topical anesthesia in a patient with deep-set eyes.

## Case presentation

A 67-year-old male presented with decreased vision in both eyes. On examination, his best-corrected visual acuity (BCVA) was 6/60 (plano) in both eyes, with normal intraocular pressure. Slit-lamp examination revealed Grade III-IV nuclear sclerosis cataracts in both eyes, while fundus examination was normal. His vital signs and systemic examination were normal. The patient was scheduled for phacoemulsification cataract surgery in the left eye. The patient also had deep-set eyes characterized by a prominent brow ridge, a prominent nasal bridge, sunken eye sockets, and a deep superior sulcus (Figure 1a, 1b), which posed significant challenges for intraocular surgery, particularly when performed from the superior (head-end) position. Facial biometric assessment showed that the depth of the nose (d), measured as the distance from the nasion (n) to the apex of the cornea (a) (n - a), was 17 mm, while the distance from the outer canthus (c) to the apex of the cornea (a) was 6 mm. The ratio of the depth of the nose (d) to the outer canthus-to-nasion distance was 0.74, which is higher than the normal value of 0.44 [1]. Additionally, the nasion-to-pronasale (n-prn) distance was 54 mm, exceeding the normal value of 44.66 ± 3.34 mm. These facial biometric parameters fulfilled the criteria for deep-set eyes for femtosecond laser docking, as described by Khokhar et al. [1]. 

**Figure 1:**
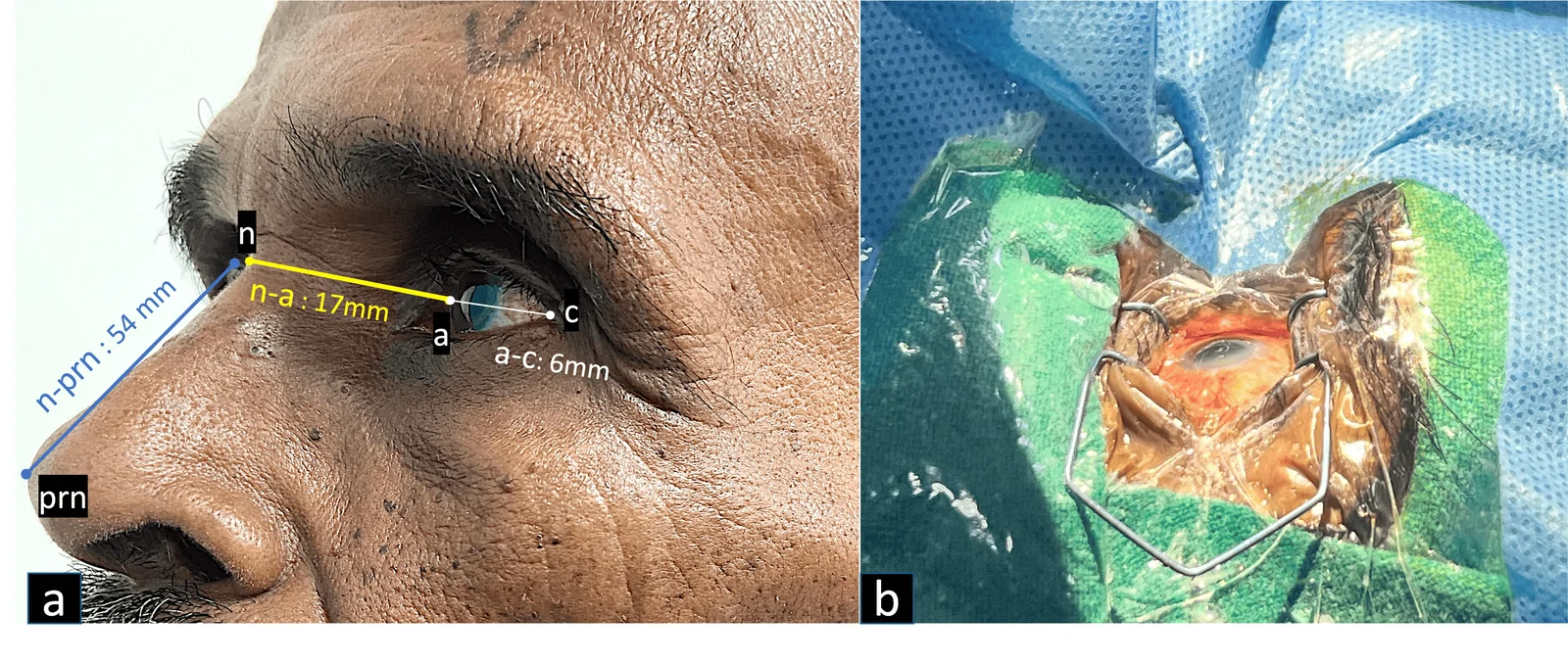
Clinical and intraoperative appearance of a deep-set eye. (1a) Clinical photograph demonstrating a deep-set eye with prominent eyebrows, a prominent nasal bridge, and a deep superior sulcus. The distance from the nasion (n) to the pronasale (prn) (n − prn) is 54 mm, the distance from the nasion (n) to the apex of the cornea (a) (n − a) is 17 mm, and the distance from the outer canthus (c) to the apex of the cornea (a) (a − c) is 6 mm. (1b) Intraoperative side-view photograph in the supine position demonstrating the deep-set globe with the prominent brow positioned anteriorly relative to the globe, resulting in limited surgical working space. Written informed consent for publication of the figure was obtained from the patient.

The patient underwent phacoemulsification cataract surgery with posterior chamber intraocular lens (IOL) implantation under topical anesthesia (Video 1). In this technique, with the surgeon in the superior (head-end) position (Figure 2a), side-port entries were created using a 15-degree keratome at the 2 o'clock position (Figure 2b) by having the patient look right and at the 10 o'clock position (Figure 2c) by having the patient look left. A bent microvitreoretinal (MVR) knife can be used to create the incision without requiring globe movement. However, in our case, we preferred a straight 15° keratome based on the surgeon's preference, which necessitated the above-mentioned right- and left-looking eye movements during incision creation. To construct the main superonasal incision at the 11 o'clock position, the patient was asked to look downward (downlook technique), providing good exposure with the incision site aligned approximately in plane with the direction of entry. By applying counter-traction using forceps inserted through the side port, the main-port incision at the superonasal position was completed using a 2.2 mm keratome (Figure 2d). Similarly, to facilitate smooth insertion of the capsulorhexis forceps, the downlook approach allowed easy insertion of the Utrata forceps (Figure 2e) to perform a continuous curvilinear capsulorhexis (CCC). Using the same downlook approach, the phaco probe was inserted (Figure 2f), and nuclear emulsification was performed (Figure 2g). Cortical matter removal was accomplished through the side ports at the 2 and 10 o'clock positions using curved irrigation and aspiration cannulas. The curved design of the cannulas, together with the adequate working space at these locations, allowed the procedure to be performed in the primary gaze without requiring the downlook approach (Figure 2h). IOL insertion was then performed using the downlook approach (Figure 2i). Side-port hydration was performed in the primary gaze without requiring the downlook approach, as the use of a bent hydro-cannula combined with adequate horizontal working space facilitated easy maneuverability in the primary gaze (Figure 2j). Hydration of the main incision was performed in the downlook position to obtain adequate superior working space (Figure 2k). On postoperative day 1, the patient achieved a BCVA of 6/6 (plano), with a clear cornea and a well-centered IOL implanted within the capsular bag (Figure 2l). 

**Video 1:** This video demonstrates a simple downlook technique , in which the patient is instructed to look downward during key steps of phacoemulsification in a deep-set eye from superior (head-end) approach under topical anesthesia. Written informed consent for publication of the video was obtained from the patient.

**Figure 2:**
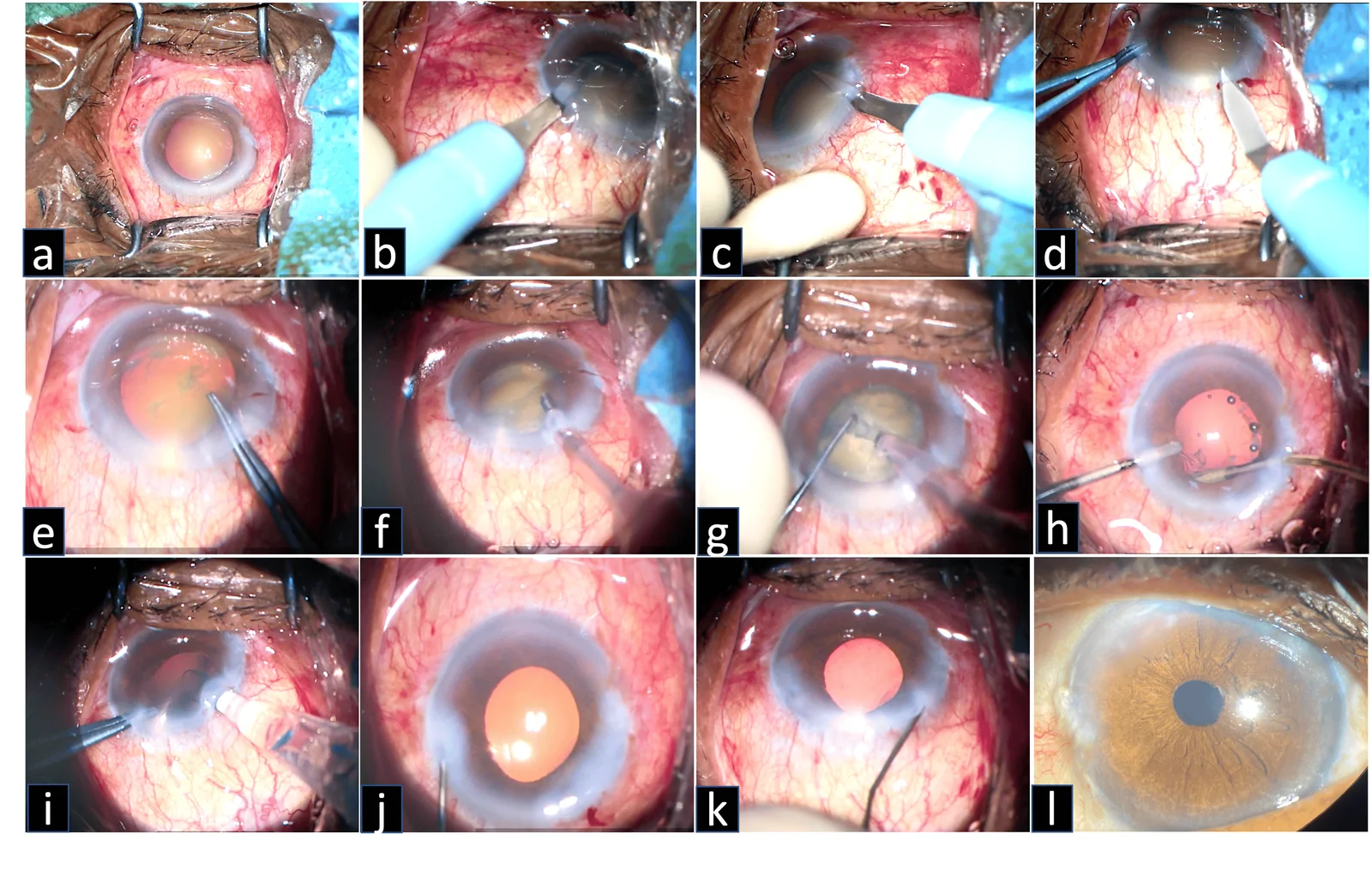
Intraoperative steps of the downlook approach for phacoemulsification in a deep-set eye. (a) Intraoperative appearance of the deep-set left eye, demonstrating the posteriorly positioned globe. (b) Patient instructed to look right for creation of the 2 o'clock side-port incision. (c) Patient instructed to look left for creation of the 10 o'clock side-port incision. (d) Downlook approach adopted to improve exposure of the superior globe, facilitating creation of the main corneal incision. (e) Continuous curvilinear capsulorhexis performed using Utrata forceps in the downlook position, providing improved superior surgical access. (f) Downlook position facilitating insertion of the phacoemulsification probe through the main incision. (g) Phacoemulsification of the nucleus performed in downlook gaze, allowing improved instrument maneuverability within the limited superior working space. (h) Cortical aspiration performed in primary gaze without requiring the downlook approach, as adequate nasal and temporal working space combined with a curved irrigation-aspiration (I/A) handpiece allows comfortable maneuverability through the side ports. (i) Foldable intraocular lens (IOL) implantation through the main incision using the downlook approach. (j) Side-port hydration performed in primary gaze without requiring the downlook approach, as the use of a bent hydro-cannula combined with adequate horizontal working space facilitates easy maneuverability. (k) Hydration of the main incision performed in the downlook position to obtain adequate superior working space. (l) Postoperative outcome showing a well-centered intraocular lens implanted within the capsular bag.

## Discussion

Deep-set eyes pose a common challenge during intraocular surgeries, particularly phacoemulsification cataract surgery, because of the limited surgical working space, especially in the superior region of the cornea. Although the temporal approach is commonly preferred, a superior approach may be indicated in patients with temporal corneal scars or diseases, superior or superonasal steep corneal astigmatism, or based on the surgeon's preference. To the best of our knowledge, no specific technique has been described in the literature [1-3] to facilitate phacoemulsification through the superior approach in patients with deep-set eyes. The Trendelenburg position and the reverse Trendelenburg position with neck extension may improve exposure of the superior globe. In these positions, tilting the patient's body with elevation of the feet or extending the neck causes the globe to rotate downward to maintain alignment with the operating microscope, thereby exposing more of the superior globe. However, these techniques have potential drawbacks, including increased intraoperative intraocular pressure and neck discomfort [2,4-8]. In contrast, our downlook approach achieves a similar effect by selectively rotating the globe downward without changing the patient's body position, thereby improving exposure of the superior globe while avoiding the physiological effects associated with Trendelenburg or reverse Trendelenburg positioning, such as changes in intraocular pressure, venous return, and patient discomfort.

The following points should be considered when performing this technique for phacoemulsification under topical anesthesia in patients with deep-set eyes: Good patient cooperation is essential and should be assessed preoperatively at the slit lamp after appropriate counseling [9]. If the patient is unlikely to cooperate, the procedure should be performed under local anesthesia, such as peribulbar anesthesia. In such cases, the downlook approach can be achieved with the assistance of fixation forceps to position the globe as required. During the procedure, frequent application of balanced salt solution (BSS) to wet the cornea should be avoided, as it may result in fluid pooling in patients with deep-set eyes, which disturbs the working area. Instead, application of 2% dispersive hydroxypropyl methylcellulose (HPMC) provides better and more sustained corneal wetting. Intraoperative assessment is essential to determine whether instrument insertion and maneuverability are adequate in the primary gaze or whether the downlook approach is required. If instrument insertion and maneuverability are adequate in the primary gaze, the procedure can be performed without globe movement. However, if instrument insertion or maneuverability is difficult, particularly in the superior or superonasal region of the globe, the eye can be gently moved into downlook as needed. The degree of downlook of the eye, as well as chin elevation, should be adjusted according to the surgeon's visualization, retroillumination, and ease of instrument maneuverability.

## Conclusions

Superior (head-end) phacoemulsification in patients with deep-set eyes can be technically challenging because of restricted access to the superior globe and limited instrument maneuverability. The downlook technique is a simple maneuver that provides improved superior corneal and globe exposure, approximating that achieved with the Trendelenburg position or reverse Trendelenburg position with neck extension, while facilitating superior main-port incision creation and intraocular instrument manipulation without altering the patient's body position. Adequate patient cooperation is essential for the successful implementation of this technique, particularly under topical anesthesia. When a superior approach is indicated, the downlook technique serves as a useful adjunct to improve surgical access and facilitate safe surgical maneuvers, while the temporal approach remains a standard alternative when appropriate. As this is a single case report, further studies involving a larger number of patients are warranted to evaluate its efficacy.

## References

[1] Khokhar S, Rani D, Rathod A (2023). Anthropometry of deep-set eyes with respect to difficulty in docking during femtosecond laser procedures. Indian J Ophthalmol.

[2] Martz TG, Karlin J, Prum BE (2018). Cataract surgery and the deep-set eye. JCRS Online Case Rep.

[3] Singh K, Misbah A, Saluja P, Singh AK (2017). Review of manual small-incision cataract surgery. Indian J Ophthalmol.

[4] Afshar AR, Pongsachareonnont P, Siegner SW, Stewart JM (2014). Trendelenburg positioning with temporal approach for vitreoretinal surgery in a patient with severe kyphosis. Eye (Lond).

[5] Awad H, Santilli S, Ohr M (2009). The effects of steep trendelenburg positioning on intraocular pressure during robotic radical prostatectomy. Anesth Analg.

[6] Jensen J, Gill N, White WA (2025). Outcomes of a novel positioning technique for ophthalmic surgery in patients with atypical anatomy. Cureus.

[7] Ripa M, Schipa C, Kopsacheilis N (2022). The impact of steep Trendelenburg position on intraocular pressure. J Clin Med.

[8] Hoshikawa Y, Tsutsumi N, Ohkoshi K (2014). The effect of steep Trendelenburg positioning on intraocular pressure and visual function during robotic-assisted radical prostatectomy. Br J Ophthalmol.

[9] Fraser SG, Siriwadena D, Jamieson H (1997). Indicators of patient suitability for topical anesthesia. J Cataract Refract Surg.

